# Supplementary material for: The limits of egg recognition: testing acceptance thresholds of American robins in response to decreasingly egg-shaped objects in the nest
Source: R Soc Open Sci. 2021 Jan 27;8(1):201615. doi: 10.1098/rsos.201615 (PMC7890492; doi:10.1098/rsos.201615)

**Hauber et al. Supplementary Electronic Material**

Supplementary Figure 1. Egg rejection responses to the two different, continuous series of model egg-shape versions with standardized predictor metrics: A: z-score transformed panel-length series and B: z-score transformed width series. The white circles represent the control model cowbird eggs (from 2019) in each plot. Shaded areas represent the 95% confidence intervals around the logistic regression curves (black lines).

A


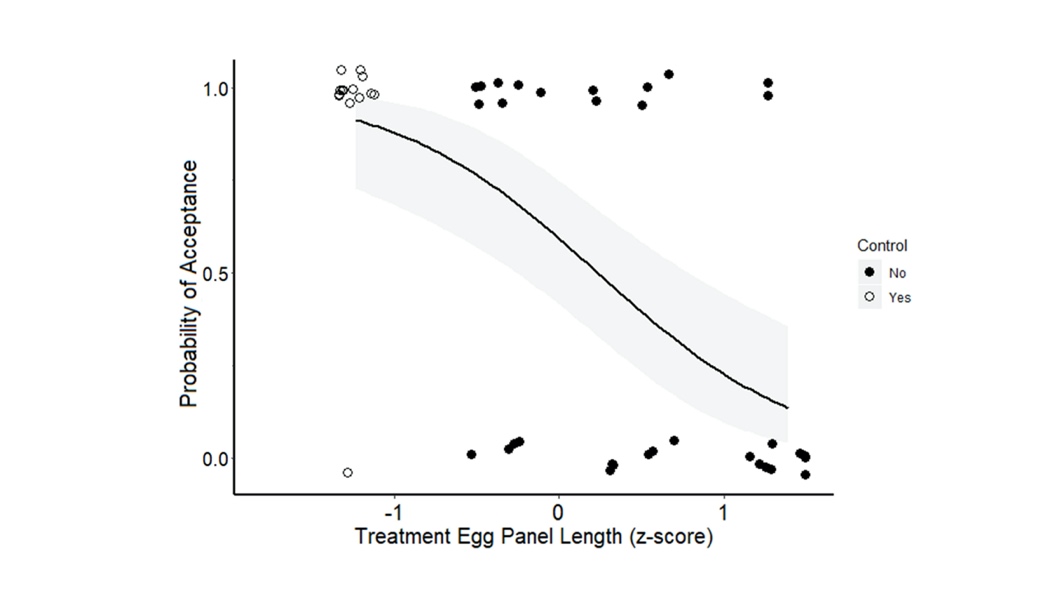


B


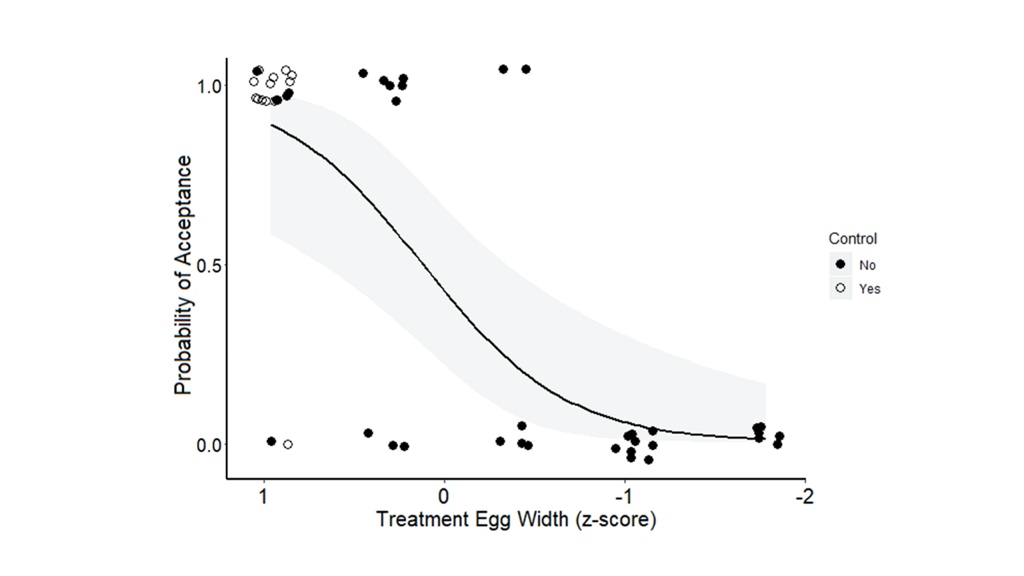

Supplement: Supplementary Figure 1. [file rsos201615supp1.docx]
